# Circular RNA triple functional domain promotes osteoarthritis’ development by modulating the microRNA-136-5p/Nicotinamide phosphoribosyltransferase axis

**DOI:** 10.1080/21655979.2021.2018095

**Published:** 2022-02-22

**Authors:** Jin Yang, Qi Li, Tingting Wang, Ke Lv

**Affiliations:** Department of Orthopedics, Affiliated Hospital of Shaanxi University of Chinese Medicine, Xianyang, Shaanxi Province, China

**Keywords:** Circtrio, miR-136-5p, NAMPT, osteoarthritis

## Abstract

Numerous studies have affirmed the participation of circular RNA (circRNA) in osteoarthritis (OA)’ development. Previous studies have exposed the elevation of the circRNA triple functional domain (TRIO) in OA, while the molecular mechanism of its effect on OA remains ambiguous. During the study, it was discovered the up-regulation of circTRIO in OA rats and interleukin-1β-treated chondrocytes. Knockdown circTRIO facilitates chondrocyte viability, but suppresses the inflammation, the apoptosis, and matrix metalloproteinases (MMP)-3 and MMP-13 expression, whereas up-regulation aggravates OA. The effect of up-regulation or under-expression of circTRIO on chondrocytes was reversed via the knockdown of Nicotinamide phosphoribosyltransferase (NAMPT) or microRNA (miR)-136-5p separately. Mechanically speaking, circTRIO competitively adsorbing miR-136-5p to target NAMPT influences OA. Briefly, the results of this study inform that the circTRIO/miR-136-5p/NAMPT axis is momentous in OA progression and is supposed to be a promising therapeutic target for some time.

## 1. Introduction

Osteoarthritis (OA) is a long-term joint illness induced by various factors, characterized by degeneration of articular cartilage, intermittent pain, and loss of mobility, seriously reducing the patient’s quality of life [1,2]. The onset of OA is mainly implicated in age. Currently, OA treatments are mainly focused on ameliorating pain while among which the loss of articular cartilage could not be altered [3,4]. In the present study, it was manifested that the extracellular matrix secreted by chondrocytes is degraded by matrix metalloproteinases (MMP), inducing inflammation [5] and chondrocyte apoptosis. Nevertheless, there is an obscure OA pathogenesis. Therefore, in-depth exploring of OA’s pathogenesis is vital for exploration of novel therapeutic targets and therapies.

Circular RNA (circRNA), a non-coding RNA molecule stably expressed in the body, can covalently bind to form a circular structure owing to the lack of a 3’-end poly (A) tail and 5’-end cap [6]. The role of circRNAs is gaining rapid attention in a great deal of illnesses, such as cancer [7], neurodegenerative illness [8], diabetes [9], obesity [10,11], etc. Recently, plenty of studies have affirmed the participation of circRNAs in the occurrence and development of OAs. For example, Zhu H *et al*. found that depletion of circ_0136474 and recovery of microRNA (miR)-766-3p alleviate oxidative damage to chondrocytes in interleukin (IL)-1β-induced osteoarthritis with interaction of DNA methyltransferase 3A (DNMT3A) [12]. Besides, Guo Z *et al*. found that exosomal-derived circBRWDD1 accelerates the development of osteoarthritis with the miR-1277/TNF receptor-associated factor 6 (TRAF6) axis [13]. CircRNA triple functional domain (TRIO), a newly discovered circRNA, is elevated in facet osteoarthritis, which was manifested in a previous study [14]. However, the potential role of circTRIO in OA is still uncertain.

The purpose of this study was to explore whether circTRIO influences the occurrence and development of OA. The knockdown of circTRIO was affirmed by depressed OA inflammation and chondrocyte apoptosis. Mechanically, circTrio, a sponge of miR-136-5p, mediates Nicotinamide phosphoribosyltransferase (NAMPT) expression, thereby facilitating OA progression.

## 2 Methods

### 2.1 Clinical samples

From April 2018 to June 2020, OA cartilage tissue was collected from 46 OA patients and 12 knee trauma patients (no history of OA or rheumatoid arthritis) from Affiliated hospital of Shaanxi University of Chinese medicine. The clinical information of the subjects is shown in Table 1. Written informed consent was signed via all of the patients. The approvement of this study was implemented by the Ethics Committee of the Affiliated Hospital of Shaanxi University of Chinese medicine according to the Helsinki Declaration.

**Table 1.** The clinical information of the subjects

Characteristics	Normal group (n = 12)		OA group (n = 46)	
Age	66.54 ± 11.92		68.33 ± 13.27	
Gender	Male	7	Male	30
	Female	5	Female	16

### 2.2 Animal experiments

Forty, 8 weeks-old Sprague-Dawley adult male rats (Beijing Wita River Laboratory Animal Technology Co., Ltd.) were raised at (24 ± 2) °C, with 50–60% humidity and 12-h light circle. After 1-week adaptive feeding, the rats were casually assigned into 4 groups: the Control, the OA, the OA+ sh-NC, and the OA + sh-circTRIO. As previously described, OA was simulated via an unstable medial meniscus (DMM) model [15] and the anesthesia in the rats was through intraperitoneally injected sodium pentobarbital (40 mg/kg) during surgery. The injection of Lentivirus (1 × 10^9^ PFU, 20 μL) was implemented into recipient rats’ knee joint 1 week behind operation in the OA + si-NC and the OA + si-circTRIO groups. The same dose of saline was received in the OA and the control groups, but in the Control group the surgery was not received. Eight weeks later, the rats were euthanized by inhaling excessive CO_2_, and the knee joints were obtained and fixed with 4% paraformaldehyde. The clone of sh-NC and sh-circTRIO (Genechem, Shanghai, China) was conducted into recombinant lentiviral vectors.

### 2.3 Hematoxylin and eosin (H&E) staining

The decalcification for rat knee joint fixation was implemented with 10% ethylene diamine tetraacetic acid-2Na until the sample became soft, followed by routine dehydration and paraffin embedding. The sample was cut into sections with 4 µm thickness with periodical dewax and wash of xylene. The staining with hematoxylin and 0.5% eosin solution (Beyotime Institute of Biotechnology, Inc.), dehydration in alcohol (ascending concentrations), and seal with a neutral resin were carried out in the sections [16]. The observation of morphology of cartilage tissues was conducted under a general microscope (Nikon Corporation).

### 2.4 Safranine O staining

Safranine O staining was performed as described before [17]. The samples were stained with fresh Wegert solution, differentiated with acidic ethanol, implemented with a staining of solid green solution (Beijing Sunshine Biotechnology Co., Ltd.) and added with the saffron O stain. Dehydration (95% ethanol, anhydrous ethanol separately), clearance (xylene), and seal (optical-resin, Ni-E; Nikon Corporation) were conducted in the samples.

### 2.5 Terminal deoxynucleotide transferase DUTP notched end labeling (TUNEL) staining

With the manufacturer’s instructions accordingly, the labeling of apoptotic cells in rat cartilage with the TUNEL assay kit (Beyotime Institute of Biotechnology, Inc.) was conducted [18]. TUNEL positive cells were counted using a microscope (Nikon). The number of apoptotic cells was calculated from 5 random fields on each slide.

### 2.6 Cell culture

The culture of the human chondrocyte-line CHON-001 (#CRL-2846) (American Type Culture Collection; Manassas, VA, US) were required in Dulbecco’s Modified Eagle’s medium (HyClone, USA) comprising 1% penicillin/streptomycin (Hyclone, USA) and 10% fetal bovine serum (Gibco, USA).

### 2.7 Cell transfection

The design and synthesis of small interfering RNA targeting circTRIO and NAMPT (si-circTRIO and si-NAMPT) and negative control (si-NC), circTRIO overexpression vector (pcDNA3.1 circTRIO) and NC (pcDNA3.1), miR-136-5p mimic/inhibitor, and its NC were via Genechem (Shanghai, China). The reagents were transiently transfected into CHON-001 cells with Lipofectamine 2000 (Invitrogen, Carlsbad, CA, US) as required by the manufacturer. The collection of the cells was then implemented for subsequent experiments.

### 2.8 OA induction via IL-1β

The stimulation of an OA *in vitro* model was conducted via the reaction of CHON-001 in each well with 10 ng/mL IL-1β (Peprotech, Rocky Hill, NJ, USA). In addition, the treatment of CHON-001 with the same dose of PBS was obtained by the control group.

### 2.9 Cell counting kit (CCK)-8 detection of proliferation

The seeding of human chondrocytes was conducted on 96-well plates at 1 × 10^5^ cells/mL and the preculture was implemented. After 24-, 48-, 72- and 96-h treatment, the incubation of each well was via 10 μL CCK-8 solution [19], with the measurement of absorbance at 450 nm with a microplate meter (Thermo Fisher, USA).

### 2.10 Flow cytometry for apoptosis detection

Cell apoptosis was detected using the fluorescein isothiocyanate (FITC) Annexin V apoptosis Assay Kit (BD Biosciences, San Jose, CA, US) [20]. The resuspension of 1 × 10^5^ cells in 1 × Annexin V Binding Buffer with 5 μL FITC Annexin V and 5 μL propidium iodide (PI) solution and the staining without light and dilution in 400 μL 1 × Annexin V binding buffer and analysis on Flow Cytometer (BD Biosciences) were done.

### 2.11 Enzyme-linked immunosorbent assay (ELISA)

The determination of IL-6, IL-1β, and Tumor necrosis factor-α (TNF-α) in rat serum and CHON-001 was applied with an ELISA kit (R&D Systems, Inc.) and according manufacturer’s requirements.

### 2.12 Reverse transcription quantitative polymerase chain reaction (RT-qPCR) for expression detection

The harvesting of tissue and cells with TRIZOL reagent (Invitrogen; Thermo Fisher Scientific, Inc.) and measurement of RNA concentration via NanoDrop 2000 were obtained. The cRNAs of circTRIO and NAMPT were synthesized via PrimeScript RT Master Mix (Takara Bio, Inc.), and that of miR-136-5p via the Revertaid First Strand Complementary DNA Synthesis Kit (Thermo Fisher Sciences, Inc.). The performance of RT-qPCR was via the miScript SYBR ®-Green PCR kit (Thermo Fisher Scientific, Inc.). U6 and glyceraldehyde-3-phosphate dehydrogenase (GAPDH) were regarded as miRNA and mRNA’s endogenous controls separately. The results were analyzed using 2^−ΔΔCt^ method. Primer sequences are manifested in Table 2.

**Table 2.** RT-qPCR primer sequences

	Primer sequence (5’ – 3’)
GAPDH	Forward: 5’- GCTCTCTGCTCCTCCTGTTC-3’
	Reverse: 5’- AAATGAGCCCCAGCCCTTCTC-3’
U6	Forward: 5’- GCTTCGGCAGCACATATACTAAAAT-3’
	Reverse: 5’- CGCTTCAGAATTTGCGTGTCAT-3’
CircTRIO	Forward: 5’- CAAGCACAGTCTTCGGAAGT −3’
	Reverse: 5’- TTCTGGTGCCTGCTTCATCT −3’
MiR-136-5p	Forward: 5’- GCCGAGACTCCATTTGTTTTGAT −3’
	Reverse: 5’- CAGTGCGTGTCGTGGAGT −3’

### 2.13 Western blot

The cells and tissues were lysed on ice with Radio-Immunoprecipitation assay lysis buffer (MA0151, MeilunBio, China) and a mixture of protease inhibitors (FD1001, FDBio, Hang, China). Protein expression was detected in sulfate polyacrylamide gel electrophoresis. Electroblot of the protein was onto a polyvinylidene fluoride membrane (Amersham Bioscience, NJ). The sealing of the membrane was required with Tris buffered saline and 5% skim milk powder, with incubation of primary antibodies and Horseradish peroxidase-conjugated secondary antibodies (FDM007 and FDR007, FDBIO). To this end, protein measurements were implemented with an enhanced chemiluminescence kit (FD8030, FDBIO). The primary antibodies were as follows: cleaved caspase-3 (9664, Cell Signaling Technology), Bax (2772, Cell Signaling Technology), Bcl-2 (12789-1-AP, ProteinTech), NAMPT (11776-1-AP, Proteintech).

### 2.14 Luciferase report experiment

The amplification and subclone of circTRIO and NAMPT 3’untranslated region (UTR) with wild- (WT) or mutant-type (MUT) miR-136-5p binding site were conducted into pMIR-GLO reporter plasmid (Promega Corporation), causing the plasmid vectors named WT/MUT-circTRIO and WT/MUT-NAMPT. There was a culture of chondrocytes (1 × 10^4^) in a 96-well plate, and introduction with the plasmid vectors and NC mimic and miR-136-5p mimic via Lipofectamine 2000 (Life Technologies; Thermo Fisher Scientific, Inc.). Finally, the measurement of luciferase activity was applied with the dual-luciferase reporter assay system (Promega Corporation).

### 2.15 Statistical analysis

The data were expressed as mean ± standard deviation (SD). GraphPad Prism 9.0 (GraphPad Software, Inc.) was applied for statistical analysis, and Student’s t-test for analysis of the differences between the two groups. A one-way analysis of variance was applied for comparisons of three or more groups. *P* < 0.05 was considered statistically significant. All data were biologically replicated at least three times.

## 3 Results

### 3.1 Knockdown circTRIO suppresses OA’s development

**Figure 1.**
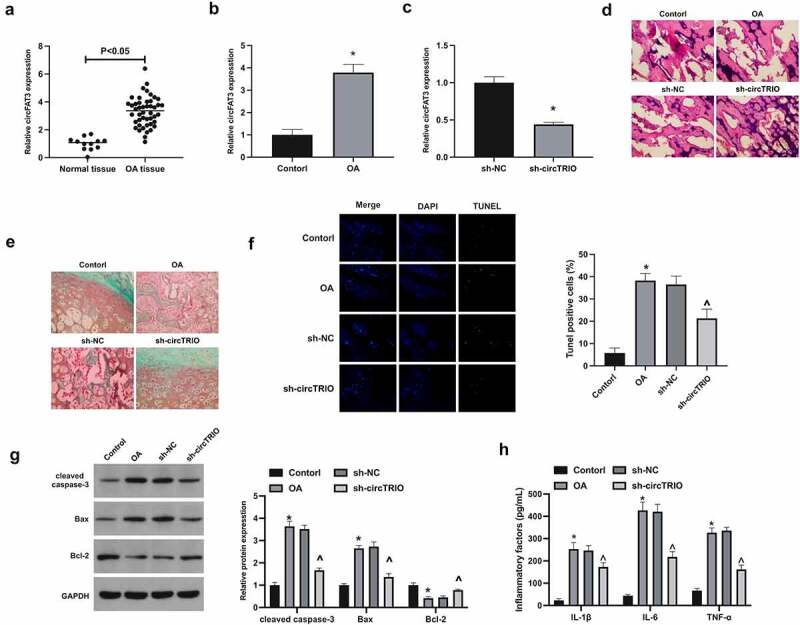
Repressed circTRIO curbs OA’s development a, b. CircTRIO in patients with OA and knee injury, and rats in the Control and the OA groups detected via RT-qPCR; c. CircTRIO in cartilage tissue of rats in the sh-NC and sh-circTRIO groups tested via RT-qPCR; d. Representative images of HE staining of rat cartilage tissue; e. Representational image of rat cartilage tissue in Safranine O staining; f. The effect of circTRIO knockdown on the apoptosis rate of rat cartilage tissue cells examined via TUNEL staining; g. The effect of circTRIO knockdown on cleaved caspase-3, Bax, and Bcl-2 in rat cartilage tested by Western blot; h. The effect of circTRIO knockdown on IL-1β, IL-6 and TNF-α in rat cartilage examined by ELISA. The values were manifested as mean ± SD (n = 10); vs the Control, *P < 0.05; vs the sh-NC, ^*P* < 0.05.

To explore the potential role of circTRIO in OA, circTRIO expression was first examined in OA (Figure 1(a,b)), affirming that circTRIO in OA patients and rats was elevated. Next, plenty of experiments have exposed that in the OA group versus the control group, for the pathological changes, articular chondrocytes in rats were disordered, and there were obvious cracks and injuries in the joints, and present severe cartilage destruction; for apoptosis in cartilage, apoptosis rate in rats, cleaved caspase-3, and Bax in cartilage tissues were up-regulated, while Bcl-2 was reduced; Inflammatory cytokines IL-1β, IL-6, and TNF-α were elevated. After knockout of circTRIO (Figure 1(c)), the joint injuries were effectively recovered (Figure 1(d)); the degree of cartilage destruction and the number of apoptotic cells in cartilage were clearly reduced (Figure 1(e,f)); Apoptotic protein expression was effectively restored (Figure 1g); Inflammatory cytokines were apparently reduced (Figure 1(h)).

### 3.2 Up-regulated circTRIO accelerates OA’s development

**Figure 2.**
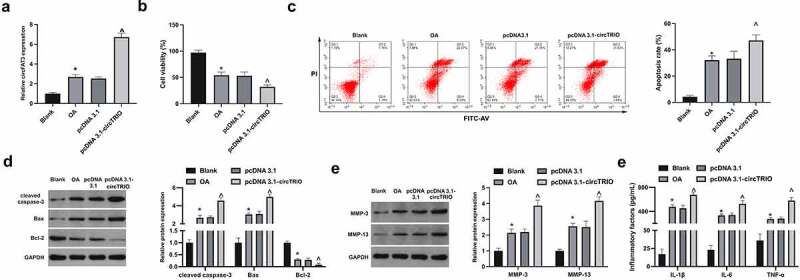
Elevated circTRIO accelerates OA’s progression a. circTRIO in CHON-001 cells after transfection of pcDNA 3.1-circTRIO detected via RT-qPCR; b. The effect of pcDNA 3.1-circTRIO transfection on CHON-001 cell viability via CCK-8; c. The effect of pcDNA 3.1-circTRIO transfection on CHON-001 apoptosis rate via Flow cytometry; d. The effect of pcDNA 3.1-circTRIO transfection on cleaved caspase-3, Bax, and Bcl-2 in CHON-001 by Western blot; e. pcDNA 3.1-circTRIO transfection’s influence on MMP-3 and MMP-13 in CHON-001 via Western blot; f. pcDNA 3.1-circTRIO transfection’s influence on IL-1β, IL-6 and TNF-α in CHON-001 by ELISA. The values were manifested as mean ± SD (n = 3); vs the Blank, *P < 0.05; vs the pcDNA 3.1, ^*P* < 0.05.

Next, the mechanism of circTRIO in OA was further explored through *in vitro* experiments. With the OA model established via chondrocytes CHON-001 treated with IL-1β, and circTRIO in the OA cell model was up-regulated by transfection. In Figure 2(a), circTRIO was clearly elevated in CHON-001 after IL-1β treatment, while up-regulation of circTRIO further enhanced circTRIO expression in CHON-001. Cell viability was then examined. As manifested in Figure 2(b), the cell viability was reduced via IL-1β treatment for 24 h to 54%, which was further declined to 32% via circTRIO up-regulation. For apoptosis detection, as affirmed in Figure 2(c), the apoptotic rate of CHON-001 was elevated via IL-1β treatment, which was further enhanced via circTRIO up-regulation. Meanwhile, examination of apoptosis-related protein expression in CHON-001 was manifested (Figure 2(d)), clarifying elevation of cleaved caspase-3 and Bax, and reduction of Bcl-2 via IL-1β treatment in CHON-001 cells. Moreover, the changes in apoptosis-related proteins were enhanced via reaction with circTRIO. MMP-3 and MMP-13 are crucial in OA’s development due to their ability to degrade matrix collagen. It was revealed that MMP-3 and MMP-13 were elevated via IL-1β treatment, which was further enhanced via up-regulation of circTRIO (Figure 2(e)). In addition, ELISA results manifested that IL-1β, IL-6, and TNF-α were augmented via IL-1β treatment (Figure 2(f)). Briefly, up-regulated circTRIO aggravates OA.

### 3.3 CircTRIO acts as a competitive endogenous (ce) RNA of miR-136-5p

**Figure 3.**
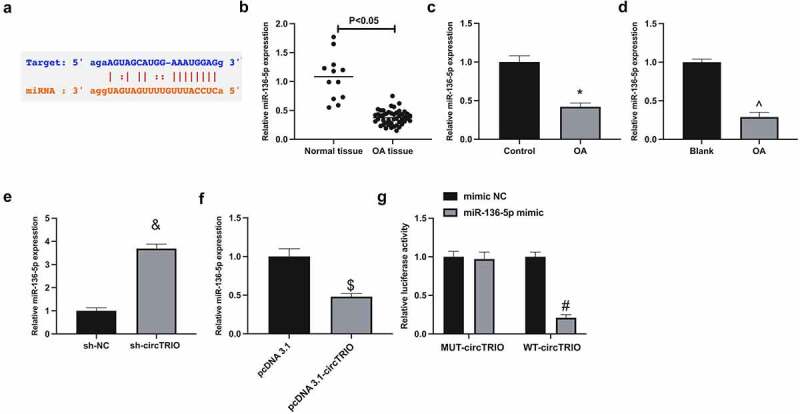
CircTRIO performs as a sponge of miR-136-5p a. The potential binding sites between circTRIO and miR-136-5p forecasted via bioinformatics website http://starbase.sysu.edu.cn; b-d. MiR-136-5p in OA patients, rats or cells detected by RT-qPCR; e, f. The effect of knockdown or up-regulation of circTRIO on miR-136-5p detected by RT-qPCR; g. The targeting relationship between circTRIO and miR-136-5p examined via the luciferase reporter assay. The values were manifested as mean ± SD (n = 3); vs the Control, *P < 0.05; vs the Blank, ^*P* < 0.05; vs the sh-NC, ^&^*P* < 0.05; vs the pcDNA 3.1, ^$^*P* < 0.05; vs the mimic, ^#^*P* < 0.05.

Next, the microRNA (miRNA) targeted via circTRIO was explored. A recent study revealed that miR-136-5p represses chondrocytic degeneration in traumatic OA [21]. Through bioinformatics website (https://starbase.sysu.edu.cn) forecasted potential-binding sites of circTRIO with miR-136-5p (Figure 3(a)). Therefore, it has been speculated that miR-136-5p might be downstream miRNA controlled by circTRIO. Subsequently, miR-136-5p was examined in OA. When exposed in Figure 3(b-d), miR-136-5p in OA patients, rats, or cells was reduced. In addition, it was conveyed that miR-136-5p could be elevated via under-expression of circTRIO, while constrained via up-regulation of circTRIO (Figure 3(e,f)). Subsequently, the targeting of circTRIO with miR-136-5p was further examined, exposing that the luciferase activity in miR-136-5p mimics introduction was repressed via WT-circTRIO, while not affected via MUT-circTRIO (Figure 3(g)). In short, circTRIO is a ceRNA for miR-136-5p.

### 3.4 circTRIO aggrandizes OA via crosstalk of miR-136-5p

**Figure 4.**
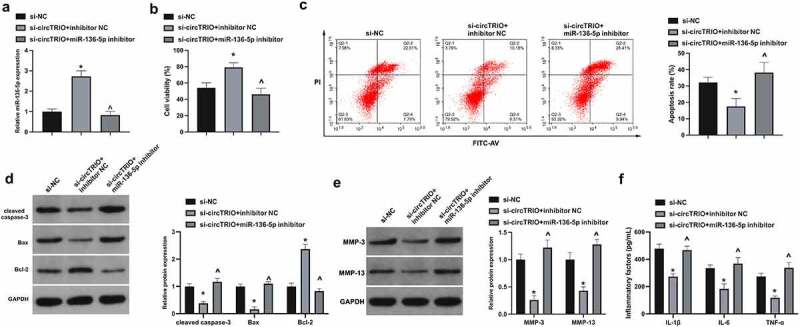
circTRIO facilitates OA via cross-interference with miR-136-5p a. Co-transfection of si-circTRIO and miR-136-5p inhibitor’s influence on miR-136-5p in CHON-001 examined via RT-qPCR; b. Co-transfection of si-circTRIO and miR-136-5p inhibitor’s influence on CHON-001 cell viability via CCK-8; c. The effect of co-transfection of si-circTRIO and miR-136-5p inhibitor on CHON-001 apoptosis rate detected by Flow cytometry; d. The effect of co-transfection of si-circTRIO and miR-136-5p inhibitor on cleaved caspase-3, Bax and Bcl-2 protein in CHON-001 by Western blot; e. Co-transfection of si-circTRIO and miR-136-5p inhibitor’s influence on MMP-3 and MMP-13 in CHON-001 via Western blot; f. Co-transfection of si-circTRIO and miR-136-5p inhibitor’s influence on IL-1β, IL-6 and TNF-α in CHON-001 via ELISA. The values were manifested as mean ± SD (n = 3); vs the si-NC, *P < 0.05; vs the si-circTRIO + inhibitor NC, ^*P* < 0.05.

Next, whether there was the involvement of miR-136-5p in OA modulated by circTRIO was investigated. The co-transfection of chondrocytes with si-circTRIO and miR-136-5p inhibitor was affirmed. It is notified that in Figure 4(a), miR-136-5p was augmented via si-circTRIO transfection. In functional assay it was clarified that CHON-001 cell viability was accelerated, whereas the apoptosis rate and cleaved caspase-3, Bax, MMP-3, MMP-13, IL-1β, IL-6, and TNF-α were restrained via si-circTRIO, which was reversed via miR-136-5p inhibitor (Figure 4(b-f)). In a word, circTRIO accelerates OA’s progression by cross-interference with miR-136-5p.

### 3.5 NAMPT is targeted through miR-136-5p

**Figure 5.**
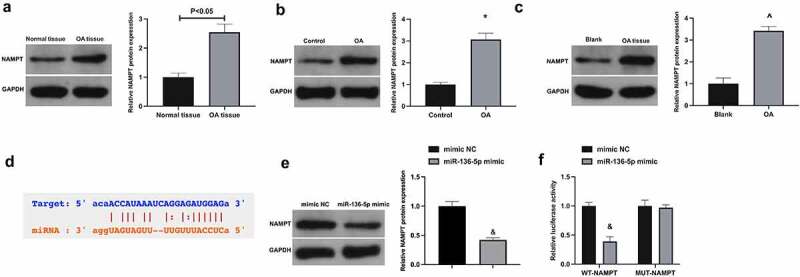
NAMPT is targeted via miR-136-5p a-c. NAMPT in OA patients, rats or cells examined by Western blot; d. The potential binding sites of NAMPT with miR-136-5p forecasted via bioinformatics website http://starbase.sysu.edu.cn; e. NAMPT in CHON-001 cells after up-regulation of miR-136-5p examined via Western blot; f. The targeting of NAMPT with miR-136-5p examined via the luciferase reporter assay. The values were manifested as mean ± SD (B, n = 10; C-F, n = 3); vs the Control, *P < 0.05; vs the Blank, ^*P* < 0.05; vs the mimic, ^&^*P* < 0.05.

MiRNAs usually bind to the 3’UTR of proteins to participate in post-transcriptional gene regulation [22]. Next, the study of target genes of miR-136-5p was manifested. NAMPT is exposed to be elevated in OA [23,24]. It was conveyed that NAMPT was up-regulated in OA (Figure 5(a-c)). Through bioinformatics website (https://starbase.sysu.edu.cn) forecasted potential-binding sites of NAMPT with miR-136-5p (Figure 5(d)). Besides, NAMPT was repressed via up-regulated miR-136-5p in CHON-001 cells (Figure 5(e)). For further verification of the targeting of NAMPT with miR-136-5p, conduction of the luciferase reporting assay was exposed to the fact that the luciferase activity of miR-136-5p mimic group was restrained via WT-NAMPT, while not changed via MUT-NAMPT (Figure 5(f)). All in all NAMPT is targeted via miR-136-5p.

### 3.6 The repression of circTRIO up-regulation on OA is weakened by downregulation of NAMPT

**Figure 6.**
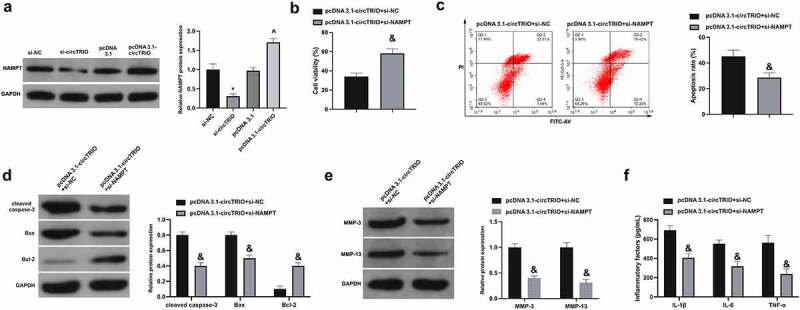
Depression of elevated circTRIO on OA is attenuated by decline of NAMPT a. The effect of knockdown or up-regulation of circTRIO on NAMPT in CHON-001 examined via Western blot; b. Co-transfection of pcDNA 3.1-circTRIO and si-NAMPT’s influence on CHON-001 cell viability via CCK-8; c. The effect of co-transfection of pcDNA 3.1-circTRIO and si-NAMPT on CHON-001 apoptosis rate via Flow cytometry; d. Co-transfection of pcDNA 3.1-circTRIO and si-NAMPT’s influence on cleaved caspase-3, Bax and Bcl-2 protein in CHON-001 via Western blot; e. Co-transfection of pcDNA 3.1-circTRIO and si-NAMPT’s influence on MMP-3 and MMP-13 in CHON-001 via Western blot; f. Co-transfection of pcDNA 3.1-circTRIO and si-NAMPT’s influence on IL-1β, IL-6 and TNF-α in CHON-001 via ELISA. The values were manifested as mean ± SD (n = 3); vs the si-NC, *P < 0.05; vs the si-circTRIO + inhibitor NC, ^*P* < 0.05.

Subsequently, whether there was the association of NAMPT in OA controlled via circTRIO was examined. It was found that NAMPT was reduced and elevated, separately, after circTRIO knockdown or increase (Figure 6(a)). Then, the function verification test was carried out. Consistent with result 2, OA’s progression was facilitated via circTRIO transfection. Meanwhile, the CHON-001 cell viability was enhanced, while the apoptosis rates of CHON-001 cells and cleaved caspase-3 and Bax, as well as MMP-3, MMP-13, IL-1β, IL-6, and TNF-α were suppressed via co-transfection of si-NAMPT (Figure 6(b-f)). In short, NAMPT is implicated in OA controlled by circTRIO.

## 4 Discussion

Chondrocyte apoptosis and articular tissue inflammation are the underlying reasons of OA [25]. In the meantime, circRNA control is crucial in the pathogenesis of OA [26]. This study manifested that circTRIO is a pathogenic gene leading to cartilage inflammation and apoptosis. In rat or chondrocyte OA models with circTRIO knockdown the inflammation and chondrocyte apoptosis were clearly reduced. Mechanically, circTRIO acts as a ce RNA of miR-136-5p in chondrocytes and targets NAMPT, thereby influencing inflammation and apoptosis of chondrocytes.

Previous studies have exposed the implications of circRNA in OA’s progression. For example, circHYBID controls acetylhyaluronic acid metabolism in OA chondrocytes through miR-29b-3p/TGF-β1 [27]. In addition, Chen S *et al*. discovered that circPDE4B prevents articular cartilage degeneration and facilitates repair as a scaffold for RIC8A and MID1 [28]. It was manifested that IL-1β treatment can influence the circRNA in chondrocytes [29,30]. Some of the new findings in the study, clarifying that circTRIO in chondrocytes was strengthened, while severe inflammation and apoptosis were induced via IL-1β. Meanwhile, the knockout of circTRIO protected chondrocyte injury by IL-1β. Notably, circTRIO, a non-coding RNA, owing to the stability in the blood, might be a circulating diagnostic factor for OA by detecting its expression.

The breakdown of the extracellular matrix homeostasis is a vital factor in OA [31]. In this study, circTRIO was associated with MMP-3 and MMP-13. Knock-downing circTRIO repressed MMP-3 and MMP-13 in chondrocytes stimulated via IL-1β while elevating circTRIO further accelerated them, thereby influencing OA progression.

To further understand the mechanism of circTRIO affecting OA, the target gene was predicted. It was exposed that circTRIO adsorbing miR-136-5p to affect NAMPT accelerated OA. Although studies have informed that miR-136-5p is vital in various diseases, the performance of miR-136-5p on OA’s development is limited [32–35]. Previous studies have reported that miR-136-5p from mesenchymal stem cell exosomes restrains the disruption of extracellular matrix homeostasis in chondrocytes by targeting ELF3 [21]. This study was informed that miR-136-5p also suppressed inflammation and apoptosis of chondrocytes and targeted NAMPT in OA. Additionally, miR-136-5p was competitively bound by circTRIO in OA and reversed the protective effect of knockdown circTRIO on chondrocytes, serving as new evidence supporting miR-136-5p as a key gene in OA’s development.

However, it is worth noting that, although this study found that circTRIO knockdown improved OA *in vivo* and *in vitro*, the circTRIO/miR-136-5p/NAMPT axis needs to be further verified *in vivo*. In addition, the results of this study only apply to DMM-induced rat models and lipopolysaccharide-induced chondrocyte models, and it is not clear whether targeting circTRIO has a similar impact in OA patients. It is worth noting that there is a great difference in the degree of disease and the possibility of reversal between the early and late developmental stages of OA. The results of this study are only applicable to the early targeted treatment of OA. Moreover, the downstream targets of NAMPT need to be further explored in subsequent studies. The results of this study suggest that the abnormal expression of NAMPT is expected to affect the expression of inflammatory signaling pathway-related proteins and apoptotic factors and further impact the development of OA, which requires to be explored in subsequent studies.

## 5 Conclusion

Taken together, the circTRIO/miR-136-5p/NAMPT axis is vital for cellular function and pathological damage in OA, and is supposed to be an underlying molecular target for OA’s alleviation or diagnosis. However, the role of miRNA or circRNA networks in diseases is extremely complex, so more molecular targets need to be explored in the future. Moreover, it is ambiguous whether the clinical effect of modulating circTRIO/miR-136-5p/NAMPT is consistent with animal or cell experiments, which is the limitation of this study.

## References

[1] Richard D, Liu Z, Cao J, et al. Evolutionary selection and constraint on human knee chondrocyte regulation impacts osteoarthritis risk. Cell. 2020;181(2):362–381.e28. 32220312 10.1016/j.cell.2020.02.057 PMC7179902

[2] Hunter D, Bierma-Zeinstra S. Osteoarthritis. Lancet. 2019;393(10182):1745–1759. 31034380 10.1016/S0140-6736(19)30417-9

[3] Schnitzer T, Easton R, Pang S, et al. Effect of tanezumab on joint pain, physical function, and patient global assessment of osteoarthritis among patients with osteoarthritis of the hip or knee: a randomized clinical trial. JAMA. 2019;322(1):37–48. 31265100 10.1001/jama.2019.8044 PMC6613301

[4] Gregori D, Giacovelli G, Minto C, et al. Association of pharmacological treatments with long-term pain control in patients with knee osteoarthritis: a systematic review and meta-analysis. JAMA. 2018;320(24):2564–2579. 30575881 10.1001/jama.2018.19319 PMC6583519

[5] Zengini E, Hatzikotoulas K, Tachmazidou I, et al. Genome-wide analyses using UK Biobank data provide insights into the genetic architecture of osteoarthritis. Nat Genet. 2018;50(4):549–558. 29559693 10.1038/s41588-018-0079-y PMC5896734

[6] Vo J, Cieslik M, Zhang Y, et al. The Landscape of Circular RNA in Cancer. Cell. 2019;176(4):869–881.e13. 30735636 10.1016/j.cell.2018.12.021 PMC6601354

[7] Yu J, Xu Q, Wang Z, et al. Circular RNA cSMARCA5 inhibits growth and metastasis in hepatocellular carcinoma. J Hepatol. 2018;68(6):1214–1227. 29378234 10.1016/j.jhep.2018.01.012

[8] Dube U, Del-Aguila J, Li Z, et al. An atlas of cortical circular RNA expression in Alzheimer disease brains demonstrates clinical and pathological associations. Nat Neurosci. 2019;22(11):1903–1912. 31591557 10.1038/s41593-019-0501-5 PMC6858549

[9] Shan K, Liu C, Liu B, et al. Circular Noncoding RNA HIPK3 mediates retinal vascular dysfunction in diabetes mellitus. Circulation. 2017;136(17):1629–1642. 28860123 10.1161/CIRCULATIONAHA.117.029004

[10] Lu D, Thum T. RNA-based diagnostic and therapeutic strategies for cardiovascular disease. Nat Rev Cardiol. 2019;16(11):661–674. 31186539 10.1038/s41569-019-0218-x

[11] Ren S, Xiong H, Chen J, et al. The whole profiling and competing endogenous RNA network analyses of noncoding RNAs in adipose-derived stem cells from diabetic, old, and young patients. Stem Cell Res Ther. 2021;12(1):313. 34051854 10.1186/s13287-021-02388-5 PMC8164820

[12] Zhu H, Zhu S, Shang X, et al. Exhausting circ_0136474 and Restoring miR-766-3p Attenuate Chondrocyte Oxidative Injury in IL-1β-Induced osteoarthritis progression through regulating DNMT3A. Front Genet. 2021;12:648709. 34093648 10.3389/fgene.2021.648709 PMC8177824

[13] Guo Z, Wang H, Zhao F, et al. Exosomal circ-BRWD1 contributes to osteoarthritis development through the modulation of miR-1277/TRAF6 axis. Arthritis Res Ther. 2021;23(1):159. 34082824 10.1186/s13075-021-02541-8 PMC8173917

[14] Chu C, Chunshuai W, Jiajia C, et al. Transcriptional information revealed differentially expressed circular RNAs in facet joint osteoarthritis. Biochem Biophys Res Commun. 2018;497(2):790–796. 29470979 10.1016/j.bbrc.2018.02.157

[15] Takahashi I, Takeda K, Matsuzaki T, et al. Reduction of knee joint load suppresses cartilage degeneration, osteophyte formation, and synovitis in early-stage osteoarthritis using a post-traumatic rat model. PloS One. 2021;16(7):e0254383. 34270585 10.1371/journal.pone.0254383 PMC8284605

[16] Wang Q, Ying L, Wei B, et al. Effects of quercetin on apoptosis and extracellular matrix degradation of chondrocytes induced by oxidative stress-mediated pyroptosis. J Biochem Mol Toxicol. 2021;undefined:e22951. 10.1002/jbt.22951 34791735

[17] Qilu W, Ning K, Xiaohui L, et al. Pirfenidone attenuates synovial fibrosis and postpones the progression of osteoarthritis by anti-fibrotic and anti-inflammatory properties in vivo and in vitro. J Transl Med. 2021;19(1):157. 33874948 10.1186/s12967-021-02823-4 PMC8054406

[18] Ansari MY, Novak K, Haqqi TM. ERK1/2-Mediated Activation of DRP1 regulates mitochondrial dynamics and apoptosis in chondrocytes.[J]. Osteoarthritis Cartilage. 2021;undefined:undefined. 10.1016/j.joca.2021.11.003 PMC8792336 34767958

[19] Huiyu Z, Yue L, BingBing W, et al. Semaphorin 3A mitigates lipopolysaccharide-induced chondrocyte inflammation, apoptosis and extracellular matrix degradation by binding to Neuropilin-1. J Bioeng. 2021;12(2):9641–9654. 10.1080/21655979.2021.1974806 PMC8810004 34821196

[20] Zihao L, Ziyu H, Zhang H, et al. Moderate-intensity exercise alleviates pyroptosis by promoting autophagy in osteoarthritis via the P2X7/AMPK/mTOR axis. J Cell Death Discov. 2021;7(1):346. 10.1038/s41420-021-00746-z PMC8580998 34759265

[21] Chen X, Shi Y, Xue P, et al. Mesenchymal stem cell-derived exosomal microRNA-136-5p inhibits chondrocyte degeneration in traumatic osteoarthritis by targeting ELF3. Arthritis Res Ther. 2020;22(1):256. 33109253 10.1186/s13075-020-02325-6 PMC7590698

[22] de Sousa Marta C, Monika G, Dobrochna D, et al. Deciphering miRNAs’ Action through miRNA Editing [J]. Int J Mol Sci. 2019 20;20(24):10.3390/ijms20246249. PMC6941098 31835747

[23] Laiguillon M, Houard X, Bougault C, et al. Expression and function of visfatin (Nampt), an adipokine-enzyme involved in inflammatory pathways of osteoarthritis. Arthritis Res Ther. 2014;16(1):R38. 24479481 10.1186/ar4467 PMC3978827

[24] Oh H, Kwak J, Yang S, et al. Reciprocal regulation by hypoxia-inducible factor-2α and the NAMPT-NAD(+)-SIRT axis in articular chondrocytes is involved in osteoarthritis. Osteoarthritis Cartilage. 2015;23(12):2288–2296. 26209889 10.1016/j.joca.2015.07.009

[25] Glyn-Jones S, Palmer A, Agricola R, et al. Osteoarthritis. Lancet. 2015;386(9991):376–387. 25748615 10.1016/S0140-6736(14)60802-3

[26] Wu Y, Lu X, Shen B, et al. The Therapeutic Potential and Role of miRNA, lncRNA, and circRNA in Osteoarthritis. Curr Gene Ther. 2019;19(4):255–263. 31333128 10.2174/1566523219666190716092203

[27] Liao H, Zhang Z, Chen H, et al. CircHYBID regulates hyaluronan metabolism in chondrocytes via HSA-miR-29b-3p/TGF-β1 axis. Mol med (Cambridge, Mass). 2021;27(1):56. 34058990 10.1186/s10020-021-00319-x PMC8165762

[28] Shen S, Yang Y, Shen P, et al. circPDE4B prevents articular cartilage degeneration and promotes repair by acting as a scaffold for RIC8A and MID1. Ann Rheum Dis. 2021;80(9):1209–1219. 34039624 10.1136/annrheumdis-2021-219969 PMC8372377

[29] Tao S, Huang J, Gao Y, et al. Small extracellular vesicles in combination with sleep-related circRNA3503: a targeted therapeutic agent with injectable thermosensitive hydrogel to prevent osteoarthritis. Bioact Mater. 2021;6(12):4455–4469. 34027234 10.1016/j.bioactmat.2021.04.031 PMC8120802

[30] Wang K, Shi Z, Dong D. CircATRNL1 protects against osteoarthritis by targeting miR-153-3p and KLF5. Int Immunopharmacol. 2021;96:107704. 33971492 10.1016/j.intimp.2021.107704

[31] Zhou X, Li J, Zhou Y, et al. Down-regulated ciRS-7/up-regulated miR-7 axis aggravated cartilage degradation and autophagy defection by PI3K/AKT/mTOR activation mediated by IL-17A in osteoarthritis. Aging (Albany NY). 2020;12(20):20163–20183. 33099538 10.18632/aging.103731 PMC7655186

[32] Xiong W, Wu L, Tang R, et al. Grape Seed Proanthocyanidins (GSPs) inhibit the development of cutaneous squamous cell carcinoma by regulating the HSA_circ_0070934/miR-136-5p/PRAF2 Axis. Cancer Manag Res. 2021;13:4359–4371. 34103991 10.2147/CMAR.S302084 PMC8179753

[33] Ou M, Hao S, Chen J, et al. Downregulation of interleukin-6 and C-reactive protein underlies a novel inhibitory role of microRNA-136-5p in acute lower extremity deep vein thrombosis. Aging (Albany NY). 2020;12(21):21076–21090. 33188660 10.18632/aging.103140 PMC7695373

[34] Han C, Fu Y, Zeng N, et al. LncRNA FAM83H-AS1 promotes triple-negative breast cancer progression by regulating the miR-136-5p/metadherin axis. Aging (Albany NY). 2020;12(4):3594–3616. 32074085 10.18632/aging.102832 PMC7066879

[35] Góralska J, Raźny U, and Polus A, et al. Enhanced GIP Secretion in Obesity Is Associated with Biochemical Alteration and miRNA contribution to the development of liver steatosis. Nutrients. 2020;12.2(2):476. 10.3390/nu12020476 PMC7071278 32069846

